# Tumor microenvironment remodeling in pancreatic cancer liver metastasis

**DOI:** 10.1016/j.iliver.2025.100209

**Published:** 2025-11-13

**Authors:** Luoyingzi Xie, Yuxi Long, Sean Lee, Fuming Xie

**Affiliations:** Institute of Hepatopancreatobiliary Surgery, Chongqing General Hospital, Chongqing University, Chongqing 401147, China; Department of Surgery, National University Hospital, Singapore 308349, Singapore; Institute of Hepatopancreatobiliary Surgery, Chongqing General Hospital, Chongqing University, Chongqing 401147, China

Pancreatic cancer (PC) ranks among the most aggressive solid tumors of the digestive system, and its comprehensive therapeutic outcomes remain limited. Liver metastasis is the most common form of distant spread in pancreatic cancer and constitutes a leading cause of mortality. In recent years, with the widespread application of technologies such as single-cell sequencing and spatial transcriptomics, the understanding of pancreatic cancer liver metastasis has evolved from a focus solely on tumor cell-autonomous behaviors toward a systematic investigation of its highly complex cellular interactions and immunosuppressive microenvironment. This includes intricate biological regulations such as immune evasion, intercellular communication, and extracellular matrix remodeling. The concerted actions of these regulatory and interactive processes ultimately establish the biological foundation for the liver as the preferred metastatic site for pancreatic cancer (Fig. 1).

**Fig. 1:**
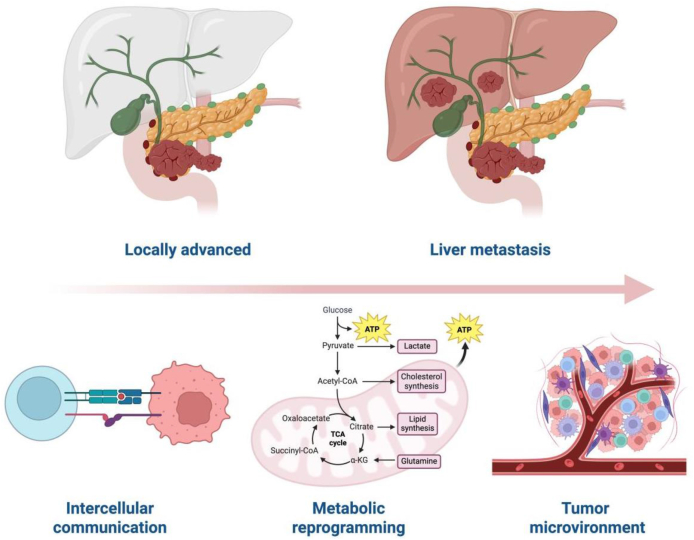
The process of microenvironment remodeling in tumor metastasis. The image was created in BioRender. Xie, F. (2025) https://BioRender.com/rm0ts57. ATP: Adenosine Triphosphate; ⍺-KG: ⍺-Ketoglutaric acid; TCA Tricarboxylic Acid; CoA: Coenzyme A.

The development of liver metastasis in pancreatic cancer primarily depends on a series of molecular alterations acquired by tumor cells themselves, which confer the capacity for migration, invasion, and colonization.^1^ Activation of epithelial–mesenchymal transition (EMT) can be triggered by exosome-mediated signal transduction. As crucial intercellular communication vehicles, exosomes can carry molecules such as integrins, which determine the organ tropism of tumor cells.^2^ Regarding tumor cell survival mechanisms, the regulation of programmed cell death pathways is particularly crucial. Studies have revealed that necroptosis, a form of regulated cell death, exerts dual regulatory roles in pancreatic cancer progression. Unlike traditional apoptosis, necroptosis actively promotes liver metastasis in pancreatic cancer by enhancing “don't eat me” signals and inducing the formation of macrophage extracellular traps, thereby expanding the understanding of the biological complexity of tumor cell death.^3^

Bidirectional communication between tumor cells and the liver microenvironment is a critical step for the successful establishment of liver metastasis. This communication is primarily achieved through exosome-mediated signaling. Research indicates that exosome-derived tRNA fragments (tRF-GluCTC-0005) can activate hepatic stellate cells, promoting the formation of a fibrotic liver microenvironment that facilitates metastatic niche formation.^4^ Similarly, the exosome-delivered CD44v6/C1QBP complex also promotes pancreatic cancer liver metastasis by driving a fibrotic liver microenvironment.^2^ The interaction between macrophages and fibroblasts represents another core aspect of liver microenvironment remodeling. Studies demonstrate that this interaction, dependent on the JAK/STAT signaling pathway, directly promotes metastatic growth of pancreatic cancer in the liver.^5^ Furthermore, the physiological process of efferocytosis (the clearance of apoptotic cells) is exploited by tumors to promote liver metastasis by reprogramming the tumor microenvironment, revealing a novel mechanism underlying the dynamic evolution of the pancreatic cancer immune microenvironment.

The immune microenvironment of pancreatic cancer liver metastasis is characterized by a significantly immunosuppressive state, maintained by various immune cell populations. Single-cell transcriptomic analyses have uncovered the accumulation and functional activation of myeloid-derived suppressor cells (MDSCs) in the liver metastatic microenvironment, which foster immunosuppression through multiple mechanisms.^4^ Notably, a subset of P2RX1-negative neutrophils with enhanced immunosuppressive capacity has been identified, closely associated with the progression of liver metastasis.^6^ Macrophages play complex and pivotal roles in the liver microenvironment. On one hand, Kupffer cells (liver-resident macrophages) show potential in preventing pancreatic cancer liver metastasis in mouse models; on the other hand, tumor-associated macrophages (TAMs) generally exert pro-tumorigenic effects.^7^ T cell dysfunction is another key feature of the immunosuppressive microenvironment. Single-cell transcriptomic profiling reveals T cell exhaustion and functional impairment within the liver metastatic niche. However, studies have also identified immune cell populations with anti-tumor potential, such as iNKT cell-mediated anti-tumor immunity, offering new directions for the development of immunotherapy strategies.^8^

The network of intercellular interactions within the liver metastatic microenvironment is exceedingly complex, involving multidirectional communication among diverse cell types. Single-cell transcriptomics and imaging mass cytometry have elucidated the spatial organization and functional relationships among different immune cell subsets. Research findings indicate that endothelial-like cancer-associated fibroblasts promote pancreatic cancer metastasis through vasculogenic mimicry and paracrine signaling, highlighting the significance of fibroblast heterogeneity.^9^ Metabolic reprogramming represents another core characteristic of the liver metastatic microenvironment. Epigenetically reprogrammed guanidinoacetate synthesis promotes pancreatic cancer metastasis and is linked to transcription-activating histone modifications, establishing a direct connection between metabolism and epigenetics. Similarly, dynamic histone lactylation could integrate metabolic, epigenetic, and immune regulation within the metastatic cascade of pancreatic cancer, forming a multidimensional regulatory network.

Despite significant progress, current research still faces numerous limitations. Issues related to sample size and representativeness are prevalent, with many studies constrained by limited sample sizes and insufficient consideration of patient heterogeneity, including variations in genetic background, tumor stage, and treatment history. At the mechanistic level, knowledge of molecular networks remains fragmented, lacking a systematic understanding of the synergistic actions of multiple molecules and pathways. Furthermore, neglecting of temporal dynamics is a notable shortcoming; most studies provide static snapshots rather than a dynamic view of the entire metastatic process, thereby limiting our understanding of metastatic evolutionary trajectories.

As a prominent domestic journal in the field of liver diseases, iLiver can leverage its influence and capacity to help establish large-scale, multicenter prospective clinical cohorts across major clinical centers in China. This would enable the systematic collection of tissue samples and clinical data from patients with pancreatic cancer liver metastasis, providing high-quality resources for translational research. Concurrently, it could establish data-sharing platforms for new technologies, such as single-cell sequencing and spatial transcriptomics, to facilitate the comparison and integration of research findings. iLiver is well-positioned to promote in-depth collaboration among multidisciplinary teams, including hepatobiliary surgery, oncology, and radiology, and to advance the comprehensive implementation of precision medicine concepts in the diagnosis and treatment of pancreatic cancer liver metastasis.

## CRediT authorship contribution statement

**Luoyingzi Xie:** Writing – original draft. **Yuxi Long:** Data curation. **Sean Lee:** Supervision. **Fuming Xie:** Conceptualization, Writing – review & editing.

## Informed consent

Not applicable.

## Ethics statement

Not applicable.

## Data availability statement

Not applicable.

## Declaration of generative AI and AI-assisted technologies in the writing process

Not applicable.

## Funding

This work was sponsored by 10.13039/501100005230Natural Science Foundation of Chongqing, China (CSTB2024NSCQ-MSX0653 to F.M.X) and Chongqing Medical Youth Leading Talent Project (YXQN202489 to L.Y.Z.X).

## Declaration of competing interest

All authors and funders have no conflicts of interest to declare.
